# Supplementary material for: Off-fault damage controls near-surface rupture behaviour in soft sediments
Source: Nat Commun. 2025 Dec 13;16:11295. doi: 10.1038/s41467-025-66467-4 (PMC12722718; doi:10.1038/s41467-025-66467-4)
Supplement: Supplementary file 1 — Supplementary Information [file 41467_2025_66467_MOESM1_ESM.pdf]

SUPPLEMENTARY INFORMATION

Off-fault damage controls near-surface rupture behaviour in soft sediments

Nicola De Paola[1]*, Rachael J. Bullock[1], Robert E. Holdsworth[1], Shmuel Marco[2], and Stefan Nielsen[1]

[1]Rock Mechanics Laboratory, Department of Earth Sciences, Durham University, South Road, DH1 3LE Durham, United Kingdom.

[2]Department of Geophysics, Tel-Aviv University, 6997801 Tel Aviv, Israel.

**Corresponding Author: Nicola De Paola. Email address: nicola.de-paola@durham.ac.uk*

Supplementary Notes – Lisan Formation and experimental simulated gouge composition

Synthetic gouges were prepared for testing by disaggregating undeformed samples of the Lisan Formation sediment collected in the field. We passed the disaggregated material through a 1 mm sieve, to remove anomalously large grains or aggregates, such as late-stage mineralisation crystals (predominantly gypsum) and fragments of fossil wood, and to ensure that all experiments had a reasonably consistent starting grain size. X-Ray diffraction (XRD) analyses of the material which passed through the sieve, and of the residual material that did not, show that their compositions are almost identical and thus the sieving process did not result in a biased sampling of finer-grained components of the sediment (Supplementary Figure 1).

The gouge is composed predominantly of aragonite and clays, which comprise between 40-60% and 30-50% of the deformed gouges, respectively, plus small amounts (up to 10%) of gypsum, quartz and calcite (Supplementary Figure 1). Separate XRD analysis of the clay fraction (<2 μm grain size) shows that it comprises mixed layer illite-smectite and kaolinite (Supplementary Figure 2). The aragonite crystals, which comprise the majority of clasts within the gouge, have a fine grain size of <15 μm and distinctive acicular habits (Supplementary Figure 3a), which are sometimes radially arranged in rosette-type structures (Supplementary Figure 3b-c). The less frequent clasts of gypsum, quartz and calcite have grain sizes up to 100 μm. The reason for using a relatively coarse 1 mm sieve was to minimise disruption and damage to individual grains and aggregates. Scanning electron microscope images of the sieved gouge confirm that the acicular aragonite crystals are un-damaged by sieving and retain their radial arrangements (Supplementary Figure 3b-c); they therefore still closely resemble the natural state of the Lisan Formation material (Supplementary Figure 3a).

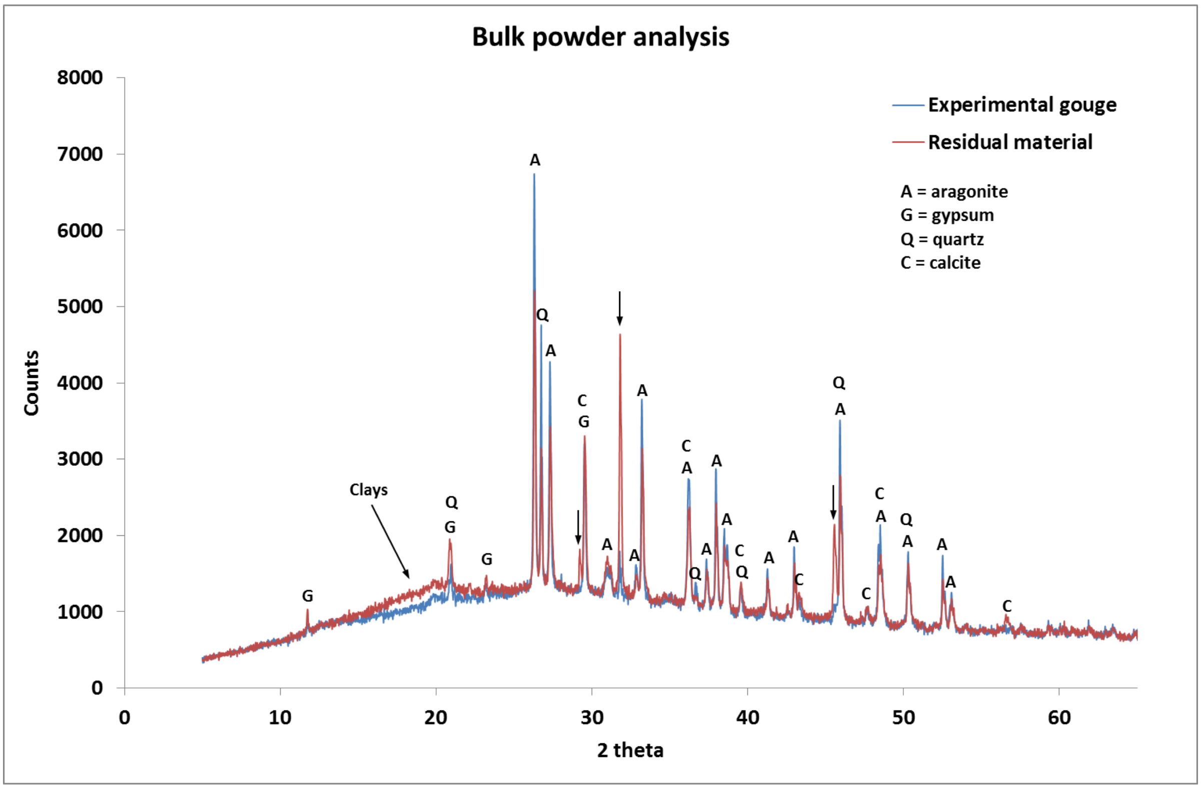

Supplementary Figure 1: XRD patterns of the sieved gouge (<1 mm) used in the experiments and the residual material, which did not pass through the sieve. *The compositions are almost identical, with the exception of the arrowed peaks, which are relatively enriched in the residual material. Possible matches of these peaks are barite and basanite. Either of these would be consistent with the residual material being enriched in late-stage crystalline mineralisation products, which one would expect to be coarser and more resistant to disaggregation than the rest of the Lisan sediment. The broad 'bump' present at low values of 2 theta is typical of clay minerals, which are poorly crystalline and so do not produce strong, clear diffractions. To identify the clays, we separated the clay fraction to produce oriented mounts (see Supplementary Figure 2).*

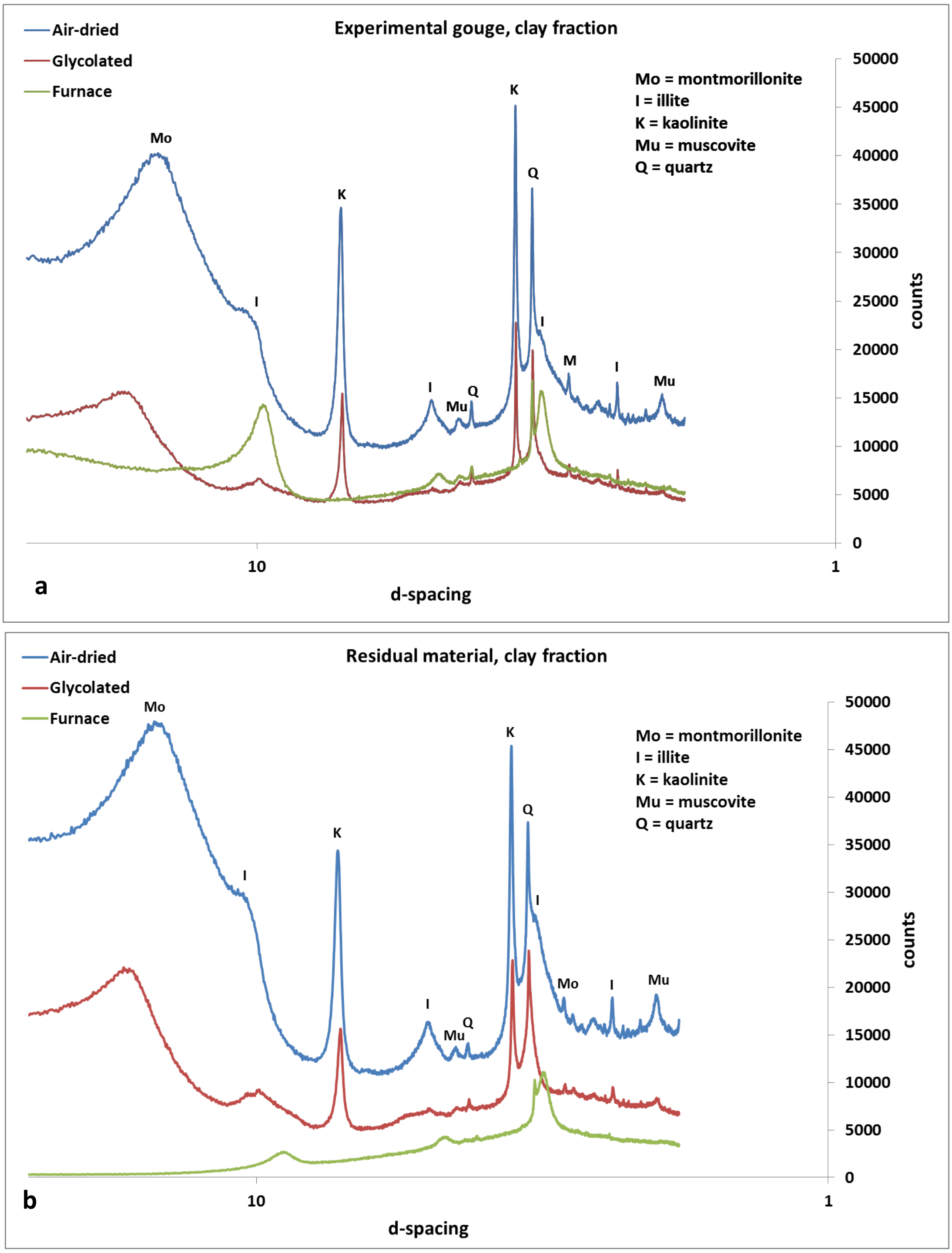

Supplementary Figure 2. a) *XRD patterns of the air-dried, glycolated and furnace-treated (550°C) clay fraction of the sieved Lisan sediment used in the experiments.* ***b)*** *XRD patterns of the air-dried, glycolated and furnace-treated (550˚C) clay fraction of the residual material, which did not pass through the sieve, and so was not used in the experiments. The composition is the same as that observed for the experimental gouge, clay fraction shown in panel* ***a****.*

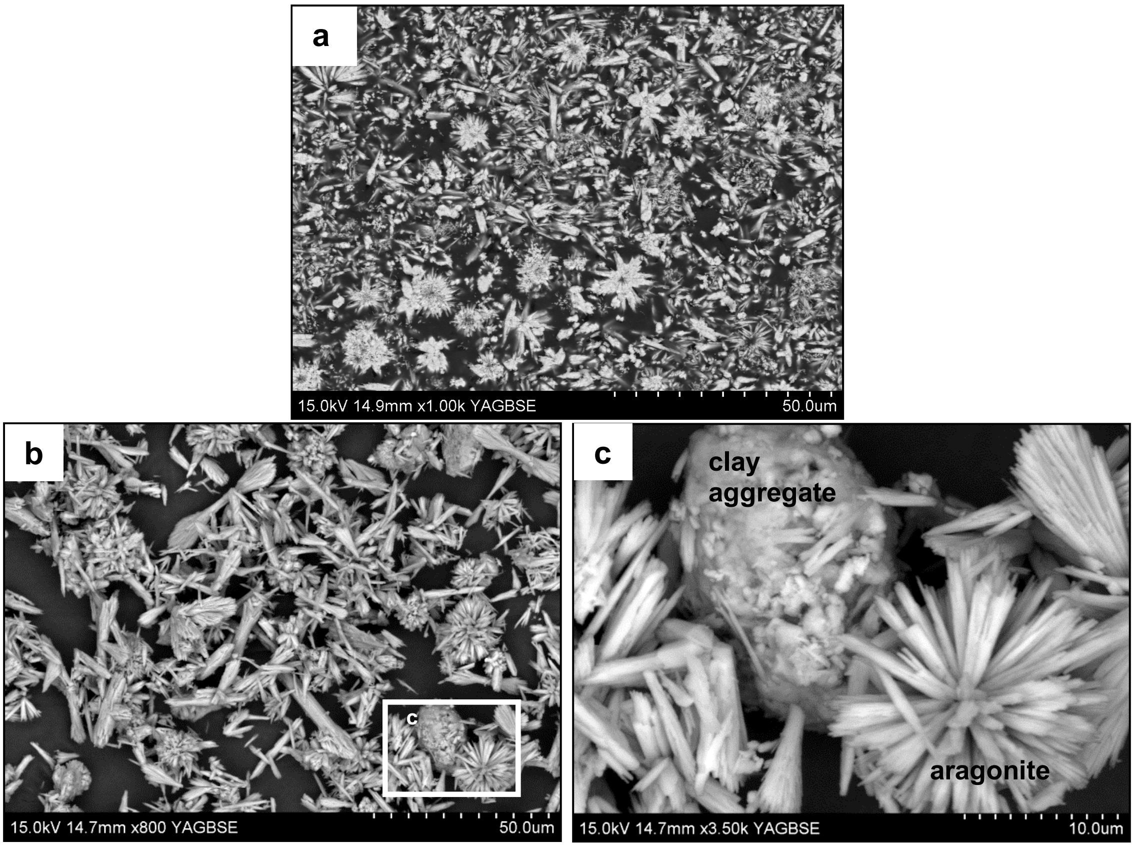

Supplementary Figure 3 – Microstructures of the natural protolith and the sieved simulated gouges used in the friction experiments. a) *SEM image of an aragonite layer from an undeformed and undisturbed sample of the Lisan Formation. The aragonite crystals are acicular with sharp, pointed terminations, and sometimes arranged in stellate or radiating aggregates, resembling rosettes.* ***b)*** *SEM image of the sieved gouge used for the experiments.* ***c)*** *Higher magnification image of the boxed area in panel* ***b****. It is evident from both panels* ***b*** *and* ***c*** *that radial arrangements of acicular aragonite crystals have retained the structures observed in the natural sample shown on panel* ***a****, so they have not been disturbed or damaged during the disaggregation and sieving process.*

Supplementary Figure 4 – The Masada fault zone (MFZ) structure

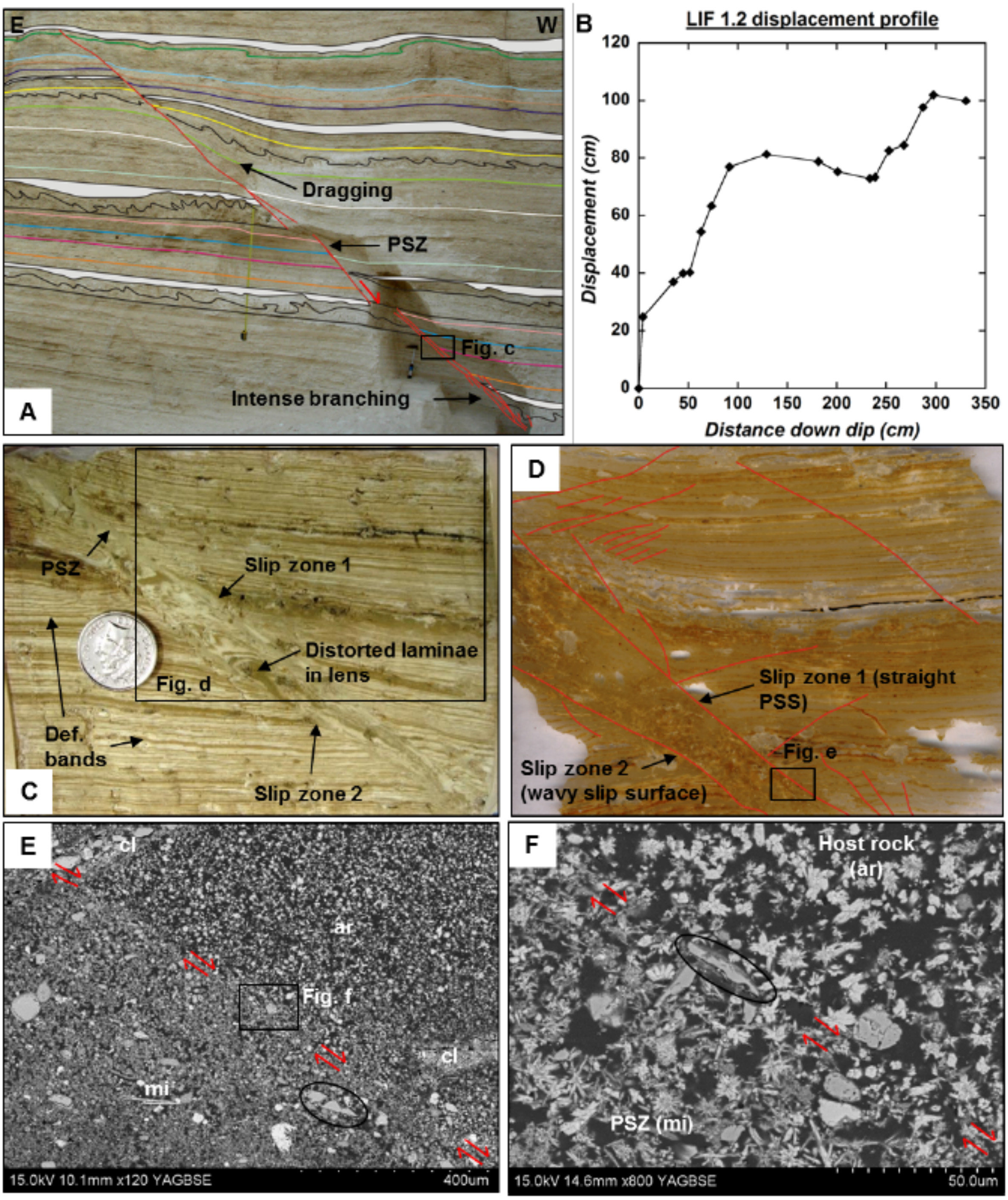

Supplementary Figure 4 – Structure and microstructures of the syn-sedimentary Masada Fault Zones. A) *Fault LIF. 1.2,* d_{max} *= 102 cm, viewed at the meso-scale in outcrop. Mixed layers are shaded grey and slumps marked in black.* ***B)*** *Displacement profile constructed for fault LIF 1.2. The plotted points correspond to the highlighted sedimentary layers in Fig. A.* ***C)*** *Hand sample from fault LIF 1.2. The location from which it is taken is shown in Fig. A. The displacement at this point is about 98 cm.* ***D)*** *High-resolution scan of thin section*

taken from boxed area in Fig. C. The slip surface bounding slip zone 1 is observed to be straighter than that bounding slip zone 2, thus we infer it to be the principal slip surface SSS. ***E)*** *SEM image of the PSS. The location from where the image is taken is shown in Fig. D. The location of the PSS is indicated by shear-sense arrows; its location is discernible due to the subtle difference in composition between the host rock, which is predominantly aragonite, and the PSZ material, which is more clay rich. A group of aligned clasts close to the PSS is circled, ar = aragonite, cl = clay-rich clastic material, mi = areas where the aragonite and clay-rich materials have mixed.* ***F)*** *Close up image of PSS and PSZ shown in Fig. E, showing the similarity in grain size of the material within the PSZ compared to the host rock. Several aragonite crystals within the slip zone maintain their concentric structures. Again some alignment of clasts along the PSS is evident (circled).*

Supplementary Figure 5 – The Masada fault zone (MFZ) structure

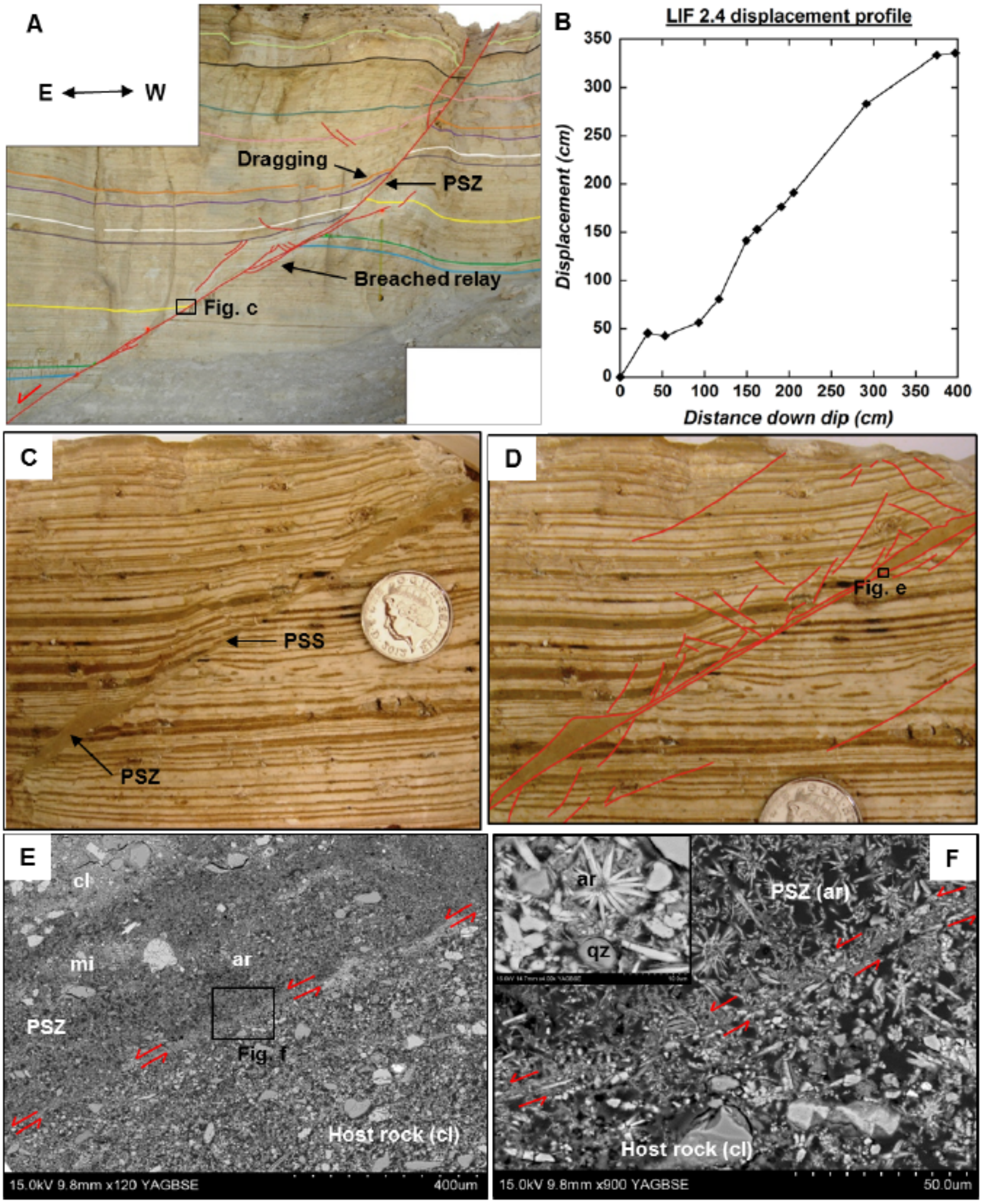

Supplementary Figure 5 – Structure and microstructures of the syn-sedimentary Masada Fault Zones. A) *Fault LIF 2.4,* D_{max} *= 337 cm, viewed at the mesoscale in outcrop.* ***B)*** *Displacement profile constructed for fault LIF 2.4. The plotted points correspond to the highlighted sedimentary layers in Fig. A.* ***C)*** *Hand sample from fault LIF 2.4. The location from which it is taken is shown in Fig. A. The displacement at this point is about 280 cm.* ***D)*** *An annotated zoomed in portion of Fig. C is shown to highlight PSZ boundaries and small scale*

off-fault damage. Again, one PSZ boundary (labelled PSS) is observed to be much straighter than the opposite PSZ boundary. ***E)*** *SEM image of the PSS. The location from where the image was taken is shown in Fig. D. The location of the PSS is indicated by shear-sense arrow; its location is discernible due to the subtle difference in composition between the host rock and the PSZ material, and an enrichment of clay along the PSS due to clay smear. The PSZ is very heterogenous in composition, e.g. note the clump of clay-rich clastic material labelled in the top left-hand side of the image, compared to the rest of the PSZ, which is aragonite rich. ar = aragonite, cl = clay-rich clastic material, mi = areas where the aragonite and clay-rich materials have mixed.* ***F)*** *Close up image of PSS and PSZ shown in Fig. E; location of PSS is indicated by shear-sense arrows and highlighted by clay smear and an alignment of acicular aragonite needles. Note how aragonite crystals in the PSZ retain their concentric arrangements of needles, showing little evidence for fracturing or comminution. Inset shows an example of an aragonite crystal and quartz (qz) clasts within the PSZ, which are surrounded by a cortex of clay.*

Supplementary Methods: Experimental apparatus specifications and sample assembly geometry.

High velocity rotary shear apparatus

Friction experiments were performed using the low to high velocity rotary shear apparatus (model MIS-233-1-77, built by the Marui & Co., Ltd. Company, Osaka, Japan) at the Rock Mechanics Laboratory, Durham University (Supplementary Figure 6). The apparatus is housed in a rigid loading frame, made of assembled steel plates, and set up in a vertical configuration, secured to the floor by a base plate (Supplementary Figure 6a-b).

Samples are mounted onto sample holders (Supplementary Figure 6c), where they are kept fixed to the main shafts and axially aligned during the experiments by a hydraulic mechanical lock (Supplementary Figure 6c). During the experiments, the upper main shaft rotates, whilst the lower one remains stationary. The upper main shaft of the apparatus is driven by an electric, servo-controlled motor with 11 kW power, 70 Nm rated torque and a maximum rotation speed of 1500 RPM. The revolving speed of the main shaft ranges from $1.5*10^{-6}$-1500 RPM, and is controlled using a system of clutches and reduction gears located in the gearbox (Supplementary Figure 6a-b). This corresponds to a peripheral slip rate of 6 cm/yr to 2 m/s, when applied to cylindrical samples with an outer diameter of 25 mm. The apparatus can accelerate to the maximum peripheral target speed of 2 m/s in 205-270 ms at normal stresses of 3 and 18 MPa, respectively, with accelerations of 3.7 and 4.9 m/s^2, respectively.

An axial load is applied to the lower main shaft by a pneumatic piston (Bellofram type cylinder) with an 82 mm stroke, and it is able to produce a thrust of 10 kN (Supplementary Figure 6a-b). The axial load system is equipped with a high-precision air regulator to reduce and automatically correct the load fluctuation during loading. Axial load measurements are performed using a thin compression load cell (strain gauge type, Supplementary Figure 6a-b) with a 10 kN capacity and a rated output of 2 mV/V ±0.5%. Axial load cell resolution is ±0.005 kN.

Torque values attained during the experiments are measured by two compression load cells (strain gauge type) (Supplementary Figure 6a-b), which are activated by a torque bar fixed to the lower main shaft. The load cell capacity is 1 kN, with a rated output of 2 mV/V ±0.5%. Torque cell resolution is $\pm5*10^{-4}$ kN.

The axial displacement values attained during the experiments are measured using a high sensitivity displacement gauge (strain gauge type) with a 10 mm capacity and a rated output of 5 mV/V ±0.1% (Supplementary Figure 6a-b). Axial displacement resolution is $\pm2*10^{-3}$ mm.

The revolution speed and the cumulative number of revolutions are measured by a tachometer and a pulse counter, respectively, based on the pulses measured by a rotary encoder with a capacity of 3600 pulses/revolution (Supplementary Figure 6a-b).

Sample assembly

A synthetic fault zone was created by sandwiching up to 1.4 g of disaggregated Lisan Formation sediment between two stainless steel cylinders (25 mm in diameter), whose ends are machined to a depth of 500 μm in a gridded fashion (Supplementary Figure 7), to simulate slip surface roughness and to ensure that slip localises within the gouge layer, rather than at the boundaries between the gouge and the steel cylinders. To limit gouge loss during the experiments, the sample assembly was confined using a Teflon ring [1], which was tightened to the steel cylinder using a hose clip (Supplementary Figure 7b). The inner edges of the Teflon rings are machined to reduce their sharpness and avoid ring damage and sample contamination by Teflon.

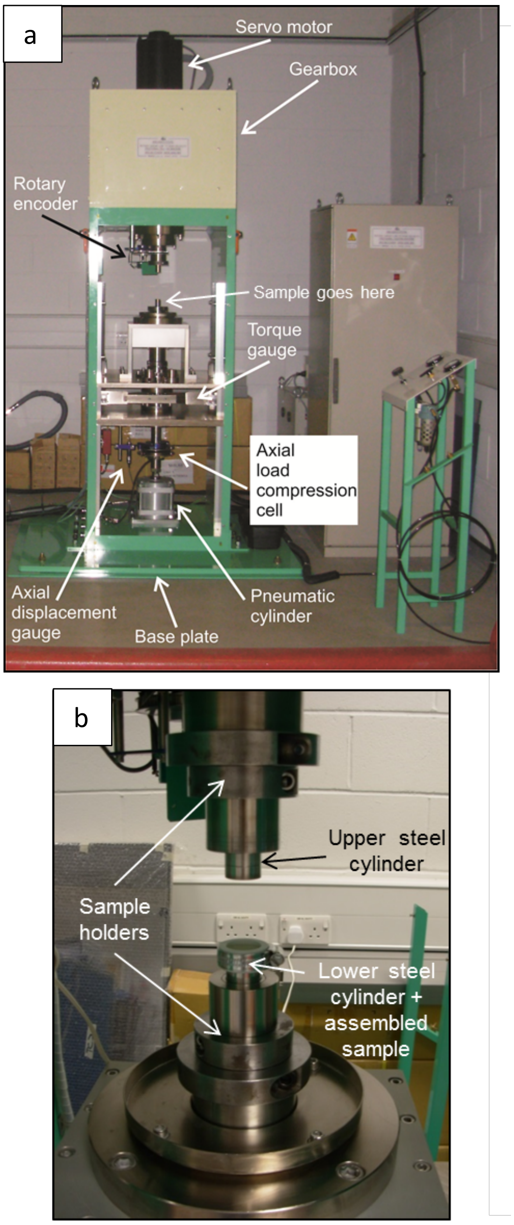

Supplementary Figure 6. a) *Photograph of the low to high velocity rotary shear apparatus installed in the Rock Mechanics Laboratory at Durham University.* ***b)*** *Close up image showing an assembled sample mounted in the apparatus.*

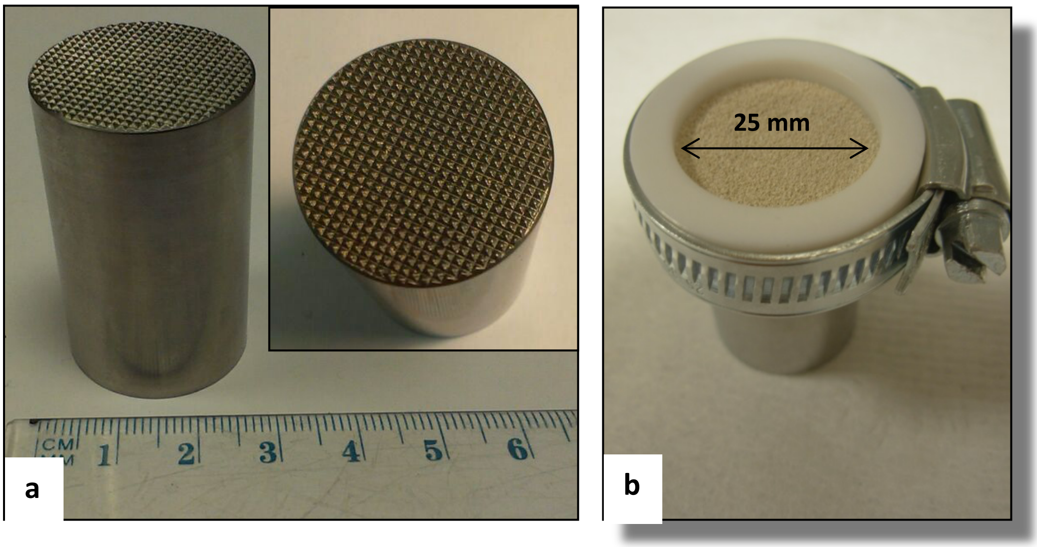

Supplementary Figure 7. a) *Stainless steel cylinders used during the experiments, whose ends are machined in a gridded fashion.* ***b)*** *Assembled gouge sample.*

Experimental determination of slip rate and slip for cylindrical samples

To calculate the slip rate within the gouge layer, it is necessary to convert angular velocity, ω (in rad/s; RPM can be converted to rad/s by multiplying by 2π/60) to linear velocity, *v* (in m/s). This can be done using the equation:

$v = \omega r$ (Eq. 1),

where *r* is the sample radius (in metres). Also, given the cylindrical shape of the samples, *v* increases with sample radius. Thus, the slip rate that we use is an "equivalent slip rate" [2,3], V_e, corresponding to the slip rate within the sample at *2/3r.* Taking into account the above factors, the equation for calculating V_e is:

$V_e = \frac{4\pi R r}{3\times 60}$ (Eq. 2),

where *R* is the revolution rate of the motor in RPM. We refer to the equivalent slip rate simply as the slip rate *v* in this thesis and, as a consequence, the, displacement, *d* is calculated as

$d = V_e t$ (Eq. 3),

where *t* is the time.

Supplementary Figure 8 – Microstructures of seismic, high velocity experiments

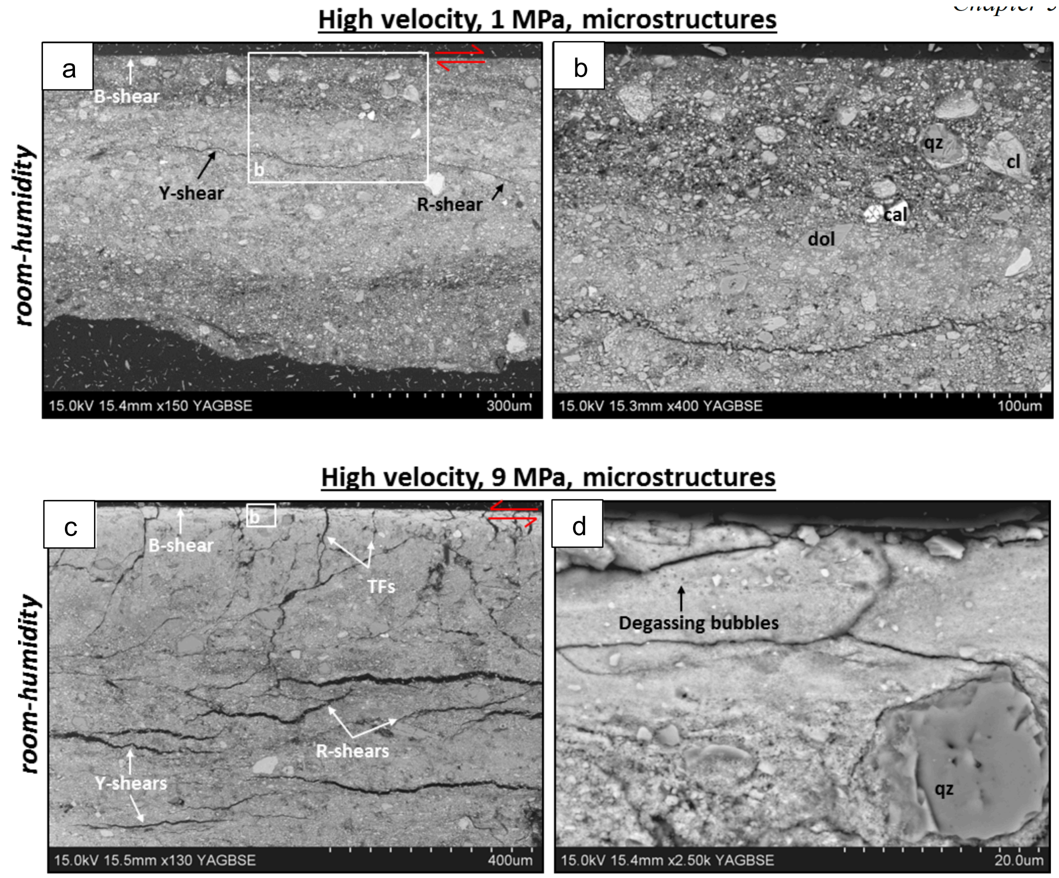

Supplementary Figure 8. *Microstructures of gouges deformed at seismic slip velocity (1.3 m s^{-1}) under 1 - 9 MPa normal stress and at room-humidity conditions. Larger clasts are labelled to aid mineral identification: cal = calcite, cl = clay-rich aggregate, dol = dolomite, qz = quartz.* ***a)*** *Room-humidity gouge (experiment 532, d = 36.77 m).* ***b)*** *High magnification image of the boxed area in fig. a.* ***c)*** *Room-humidity gouge (experiment 624, d = 4.6 m). TFs = tensile fractures.* ***d)*** *High magnification image of the boxed area in fig. c.*

Supplementary Figure 9 – High velocity friction experiments

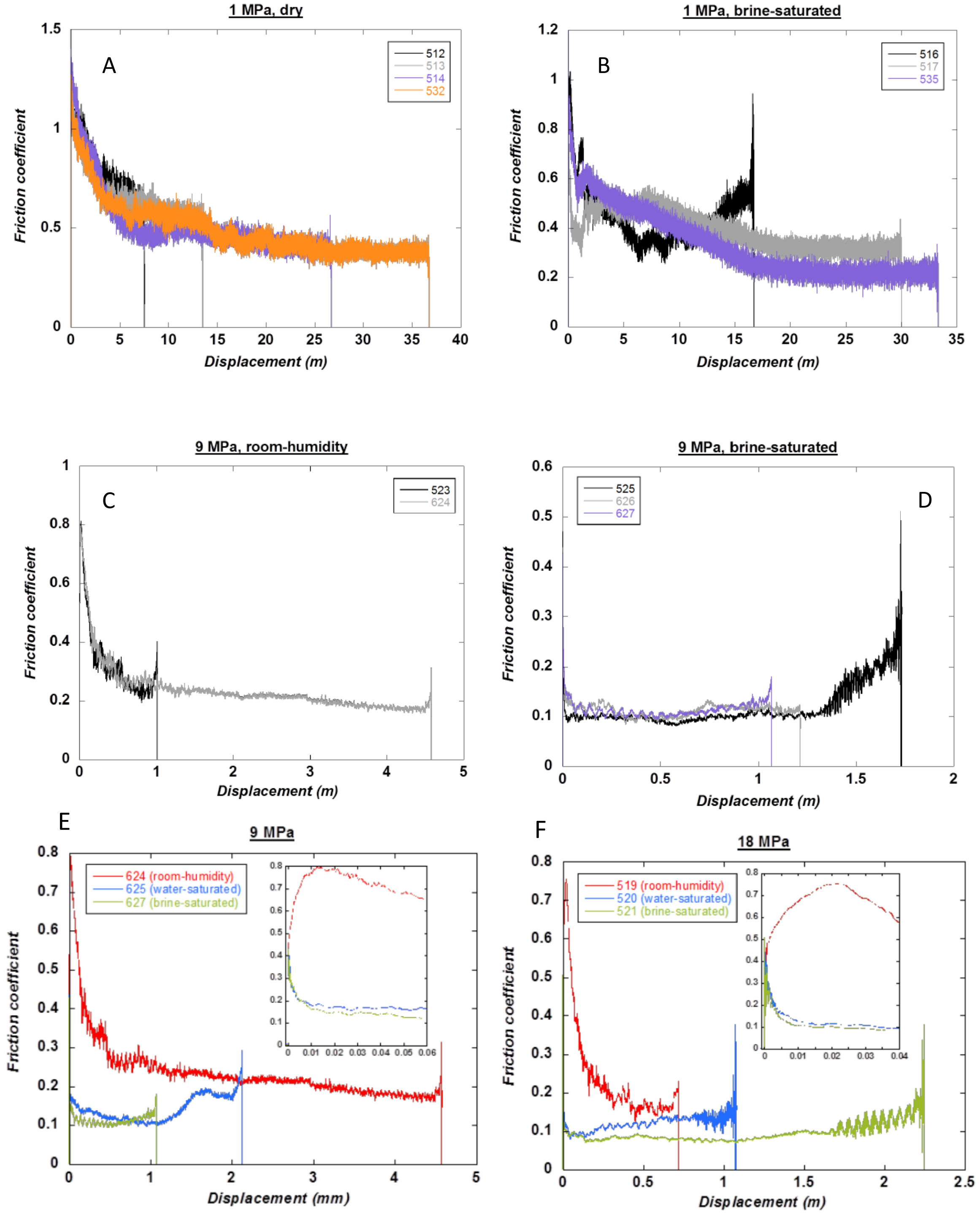

Supplementary Figure 9. *Friction evolution of room-humidity and brine-saturated gouges included in Supplementary Table I, which were deformed at 1.3 m* s^{-1} *under 1 MPa (A-B), 9 MPa (C-E) and 18 MPa (E) normal stress. The two insets in panels E – F also show the initial path of friction evolution to peak values for both dry and brine-saturated samples. The error in friction measurements depends on the level of noise in each experiment and, in general, is comprised between 0.01 and 0.03*

Supplementary Figure 10 – Energy dissipation during different amounts of fault slip

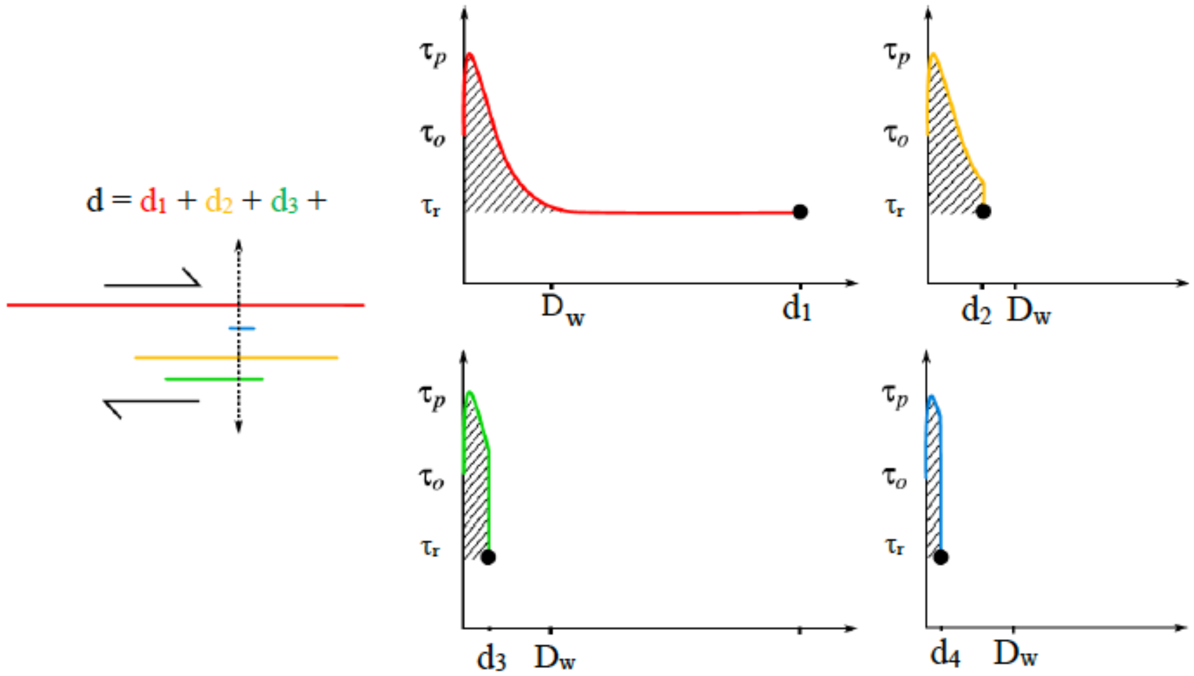

Supplementary Figure 10. *The area of the triangle, highlighted under the slip-weakening curve shown in red, represents the energy dissipated in the creation of a single shear fracture with an amount of slip* $d_1 \geq D_W$. *The areas of the trapezoid, highlighted under the slip-weakening curves shown in yellow* (d_2)*, green* (d_3) *and bleu* (d_4)*, represent the energy dissipated in the creation of a single shear fracture with an amount of slip* $d < D_W$.

Supplementary Table 1

**Exp.**	***σ_n (MPa)***	***μ_p***	***μ_r****	***μ_{ss}***	***D_f (m)***	***D_w (m)***	***E_G^{PSZ} (MJ m^{-2})***
512 (RH)	1	1.14	0.62	-	7.53	-	1.96
513 (RH)	1	1.13	0.54	-	13.46	-	3.97
514 (RH)	1	1.28	-	0.40	26.68	19.2	8.45
532 (RH)	1	1.23	-	0.39	36.77	21.3	8.95
516 (BS)	1	0.94	-	-	16.72	-	-
517 (BS)	1	0.72	-	0.32	30.02	16.7	3.34
535 (BS)	1	0.85	-	0.22	33.35	16.5	5.20
523 (RH)	9	0.81	-	0.23	1.01	0.56	1.46
624 (RH)	9	0.79	-	0.20	4.58	1.67	4.43
525 (BS)	9	0.30	-	0.10	1.73	0.01	0.01
626 (BS)	9	0.35	-	0.11	1.21	0.05	0.05
627 (BS)	9	0.36	-	0.10	1.07	0.06	0.07
519 (RH)	18	0.75	-	0.16	0.72	0.29	1.54
521 (BS)	18	0.29	-	0.09	2.25	0.03	0.05

Supplementary Table 1. *Summary of high velocity friction experiment results. (RH) = Room humidity conditions; (BS) = Brine saturated drained conditions;* σ_n = Normal stress; μ_p *= Peak friction;* μ_{ss} *= Steady-state friction;* D_f *= Final amount of slip;* D_W *= Slip weakening distance (See main text and Supplementary Information III for details);* E_G^{PSZ} *= Experimental Principal slip zone fracture energy calculated using Equation 2 in Supplementary Method A – Estimation of Fracture Energy.*

Supplementary Table 2

LIF 1.1 - Hanging-wall damage zone - Hybrid shear fractures					
Distance (cm)	*Width (mm)*	*Length (cm)*	*Orientation*	*Displacement (mm)*	*Fracture energy (MJ m^-2)*
0	10	120	001/72	30	0.11655
60	1	80	328/71	1	0.005135
130	5	300	342/70	20	0.0857
165	10	350	110/68	1	0.005135
175	5	150	340/70	1	0.005135
255	2	320	124/88	3	0.015105
286	1	25	345/75	3	0.015105
333	1	140	130/83	2	0.01017
366	1	160	330/65	2	0.01017
590	1	220	145/87	1	0.005135
675	1	300	140/65	2	0.01017
1493	10	550	328/86	20	0.0857

LIF 1.1 - Hanging-wall damage zone - Disaggregation shear bands					
Distance (cm)	*Width (mm)*	*Length (cm)*	*Orientation*	*Displacement (mm)*	*Fracture energy (MJ m^-2)*
0	1	20	116/61	30	0.11655
30	1	50	253/39	20	0.0857
35	1	40	254/38	10	0.04735
50	1	15	104/60	10	0.04735
60	1	100	240/40	45	0.145575
60	1	30	265/32	8	0.03868
60	1	10	250/32	30	0.11655
60	1	10	090/45	18	0.07893
272	2	50	330/52	10	0.04735
290	10	50	320/50	70	0.1551

LIF 1.1 - Foot-wall damage zone - Hybrid shear fractures					
Distance (cm)	*Width (mm)*	*Length (cm)*	*Orientation*	*Displacement (mm)*	*Fracture energy (MJ m^-2)*
0	3	120	335/84	3	0.015105
155	20	250	046/84	65	0.1551
180	2	400	036/85	70	0.1551
205	1	30	265/90	2	0.01017
220	3	350	002/80	6	0.02961
270	1	50	023/39	1	0.005135
290	2	40	328/72	2	0.01017
330	1	40	335/79	1	0.005135
360	1	15	026/86	1	0.005135
395	10	160	286/90	2	0.01017
413	8	350	010/86	3	0.015105
535	15	220	096/82	10	0.04735
535	15	100	096/82	10	0.04735
535	15	120	078/88	10	0.04735
535	15	70	078/88	10	0.04735
540	1	25	274/70	3	0.015105

LIF 1.1 - Foot-wall damage zone - Disaggregation shear bands					
Distance (cm)	*Width (mm)*	*Length (cm)*	*Orientation*	*Displacement (mm)*	*Fracture energy (MJ m^-2)*
0	1	30	043/80	65	0.1551
0	1	40	209/10	65	0.1551
25	20	25	194/35	25	0.102125
25	1	6	204/42	14	0.06419
45	1	10	236/70	6	0.02961
50	1	4	235/60	1	0.005135
55	2	50	186/40	90	0.1551
90	20	40	239/45	70	0.1551
145	1	30	257/30	80	0.1551
145	1	30	277/20	10	0.04735
155	1	50	240/44	80	0.1551
205	1	70	204/42	25	0.102125
265	1	15	075/36	9	0.043065
290	1	20	060/45	15	0.068025
300	1	20	082/49	20	0.0857
305	1	20	080/50	15	0.068025
305	1	20	054/55	14	0.06419

305	1	20	054/55	14	0.06419
310	1	25	088/44	10	0.04735
315	1	15	084/43	32	0.12112
343	1	15	216/66	3	0.015105
375	10	15	220/22	120	0.1551
380	2	40	199/09	180	0.1551
385	1	10	213/22	25	0.102125
385	1	10	208/17	40	0.1374
385	1	15	205/35	1	0.005135
385	1	40	195/12	1	0.005135
395	1	15	208/37	15	0.068025
445	1	15	060/60	40	0.1374
425	1	30	052/60	240	0.1551

Supplementary Table 2. *Structural data collected along a transect in the 16 m wide damage zone of fault LIF1.1, which are used to calculate the off-fault fracture energy ($E_G^{Off\text{-}fault}$) required to create hybrid shear fractures and disaggregation bands in the damage zone (See Methods A in the main text and Supplementary Figure 10 for details about the calculations). Measurements errors of damage zone fractures and their attributes in the field is < 1 mm.*

Supplementary References:

1) Mizoguchi, K., Hirose, T., Shimamoto, T., Fukuyama, E. Reconstruction of seismic faulting by high-velocity friction experiments: An example of the 1995 Kobe earthquake. *Geophys. Res. Lett.* **34**, L01308 (2007).
2) Hirose, T. & Shimamoto, T. Growth of molten zone as a mechanism of slip weakening of simulated faults in gabbro during frictional melting. *J. Geophys. Res-Sol. Ea.* **B05202**, doi:10.1029/2004JB003207 (2005).
3) Shimamoto, T. & Tsutsumi, A. A new rotary-shear high-speed frictional testing machine: Its basic design and scope of research (in Japanese with English abstract). *Journal of the Tectonic Research group of Japan* **39**, 65-78 (1994).
